# Investigation on the Degradation Mechanisms and Mechanical Property Evolution of 3D-SiO_2_-Fiber-Reinforced Polyimide Composites After Thermal Aging

**DOI:** 10.3390/polym18202556

**Published:** 2026-10-17

**Authors:** Minglong Yang, Yadan Wu, Jiahao He, Qiujin Gu, Haitao Liu

**Affiliations:** Science and Technology on Advanced Ceramic Fibers and Composites Laboratory, College of Aerospace Science and Engineering, National University of Defense Technology, Changsha 410073, China; minglongyang@126.com (M.Y.);

**Keywords:** polyimide-resin-based composite, 3D reinforcement, interface binding strength degradation, long-term thermal stability, thermal degradation mechanism

## Abstract

Understanding the thermal aging behavior of SiO_2f_/PI composites is crucial for ensuring their reliability and longevity in high-temperature aerospace applications. In this study, the thermal aging behavior of three-dimensional quartz-fiber-reinforced polyimide (3D-SiO_2f_/PI) composites was investigated. The composites were fabricated through a modified prepreg compression molding (PCM) method, including a vacuum-assisted impregnation process followed by thermal compression molding. Long-term thermal aging was carried out to evaluate the high-temperature aging resistance. The flexural properties were tested with a three-point bending test method. The micromechanical characterization was performed though nanoindentation. Interfacial properties were evaluated through single-fiber push-in tests, and the interfacial shear strength (IFSS) was calculated. The thermal aging experiment results revealed that the composites maintained excellent long-term thermal stability. The PI matrix showed no detectable oxidation or thermal degradation of after 100 h aging at 350 °C, yet no significant porosity or cracks were observed for the composite. However, further prolonged thermal exposure led to significant mechanical property degradation. The flexural strength retention rates dropped to below 15% after 300 h at 350 °C or 100 h at 400 °C aging. On the other hand, 350 °C isothermal aging for 300 h led to a progressive decrease in IFSS from 128.6 MPa to near-zero. The degradation mechanism was primarily attributed to two factors: increased porosity due to thermal decomposition of the PI matrix and significant deterioration of fiber–matrix interfacial bonding strength. These findings provide critical insights into the durability prediction and failure mechanisms of 3D-SiO_2f_/PI composites in high-temperature usage environments.

## 1. Introduction

Quartz-fiber-reinforced polyimide (SiO_2f_/PI) composites combines the exceptional thermal stability of quartz fibers (exceeding 1200 °C) and the outstanding mechanical properties and chemical resistance of polyimide resin [1,2]. These superior properties render it particularly valuable for critical applications in aerospace (e.g., fuel storage tanks) and tele-communications (e.g., 5G antenna housings) [3,4]. However, prolonged exposure to high temperature, hygrothermal conditions, or radiation environments can induce degradation of SiO_2f_/PI composites through thermal-oxidative decomposition and interfacial de-bonding, ultimately leading to mechanical performance deterioration [5,6,7]. Therein, research on long-term heat resistance has become a pivotal direction for improving the long-term reliability of SiO_2f_/PI composites.

Heat resistance determines the evolution of thermally triggered structural damage, and understanding how it drives mechanical attenuation is the prerequisite for exploring long-term thermal stability. Researchers have explored the heat resistance of SiO_2f_/PI composites and the mechanisms governing temperature-induced changes in mechanical properties. Zuo et al. investigated the tensile and bending properties of SiO_2f_/PI composites under room temperature and 250 °C [8]. Compared with the mechanical properties in room temperature, the tensile strength of SiO_2f_/PI composites remained relatively stable, whereas their bending strength decreased significantly at elevated temperatures. Additionally, Xiao et al. studied the strength retention ratio of SiO_2f_/PI composites at 400 °C. The retention rate of tensile strength at high temperatures reached 68%, while those of flexural strength and interlaminar shear strength were 58.5% and 61%, respectively [9]. To further reveal how temperature induces the degradation of mechanical properties in SiO_2f_/PI composites, Shen et al. quantified the elevated-temperature micromechanical properties of the composites using the fiber push-in test [10]. They demonstrated that the PI matrix exhibited linear softening at elevated temperature (350 °C), and the shear strength of the fiber/matrix interface decreased from 147 MPa at 25 °C to 40 MPa at 300 °C. Later, Chen et al. analyzed the temperature effect on both macroscale and microscale mechanical properties of SiO_2f_/PI composites [11]. They indicated that the interfacial shear strength between the fiber and the matrix was the fundamental factor governing mechanical property degradation. Nevertheless, these conventional evaluations of heat resistance cannot fully reflect the progressive degradation of composites subjected to prolonged thermal exposure, which necessitates research on long-term heat resistance.

Limited research has been conducted on thermal aging behavior of SiO_2f_/PI composites recently. Lu et al. showed that SiO_2f_/PI composites exhibit relatively high retention of mechanical properties owing to the protective effect of the matrix on the fibers. After thermal aging at 300 °C for 150 h, the retention rates of tensile strength tested at room temperature and 300 °C exceeded 76% and 92%, respectively, while those of flexural and interlaminar shear strengths were both greater than 85% [12]. In addition, Dong et al. indicated that the retention rate of flexural strength of SiO_2f_/PI composites was 70% after thermal aging at 230 °C for 1000 h, and they revealed that the combined effects of interfacial failure and stress accumulation induced the degradation of mechanical properties [13]. Recently, Cheng et al. investigated the temperature-dependent interfacial degradation of SiO_2f_/PI composites and revealed the macroscopic-to-atomistic thermal deterioration behaviors of the SiO_2f_/PI composite interface via a combined investigation of in situ single-fiber push-out tests and reactive molecular dynamics simulations [14]. However, there remains a lack of in situ characterization on microstructural evolution including interfacial chemical bond rupture and molecular chain reorganization. More importantly, in the reported research, either the aging temperature is not high enough (below 300 °C) or the aging duration is not long enough. Further thermal aging property research with higher temperature and longer duration in this aspect is therefore essential to facilitate the engineering application of SiO_2f_/PI composites in extreme environments, which constitutes the objective of the current study.

In this research, three-dimensional quartz-fiber-precursor-reinforced polyimide composite (3D-SiO_2f_/PI) was fabricated with a vacuum-assisted impregnation and hot-press molding method. More importantly, the prolonged high-temperature thermal aging test was carried out for the as-prepared 3D-SiO_2f_/PI composite, including the macroscopic and microscopic mechanical property degradation after 250 °C to 400 °C and a maximum of 300 h of thermal aging. Their microstructural evolution and mechanical properties (both micro- and macroscale) were systematically investigated before and after thermal aging to reveal the mechanisms behind the changes in mechanical behavior. This work about 3D-SiO_2f_/PI composites provides a novel lightweight, high-temperature-resistant, and high-strength fiber-reinforced polyimide matrix composite solution for next-generation aerospace applications.

## 2. Materials and Methods

### 2.1. Chemicals and Materials

The quartz fiber cloth (grade: QWB-2022-139, 99.99% SiO_2_ content, 241.56 g/m^2^ areal density, 0.25 mm thickness) and quartz yarn (195 Tex) were purchased from Filhua Quartz Glass Co., Ltd. Jingzhou, Hubei, China. The phenylacetylene-terminated polyimide resin (PMR350, 47% concentration with ethanol solvent) was purchased from Ningbo Institute of Materials Technology & Engineering. Other necessary auxiliary chemical reagents (such as analytical-grade ethanol, acetone, etc.) were purchased locally.

### 2.2. Material Preparation

**Figure 1:**
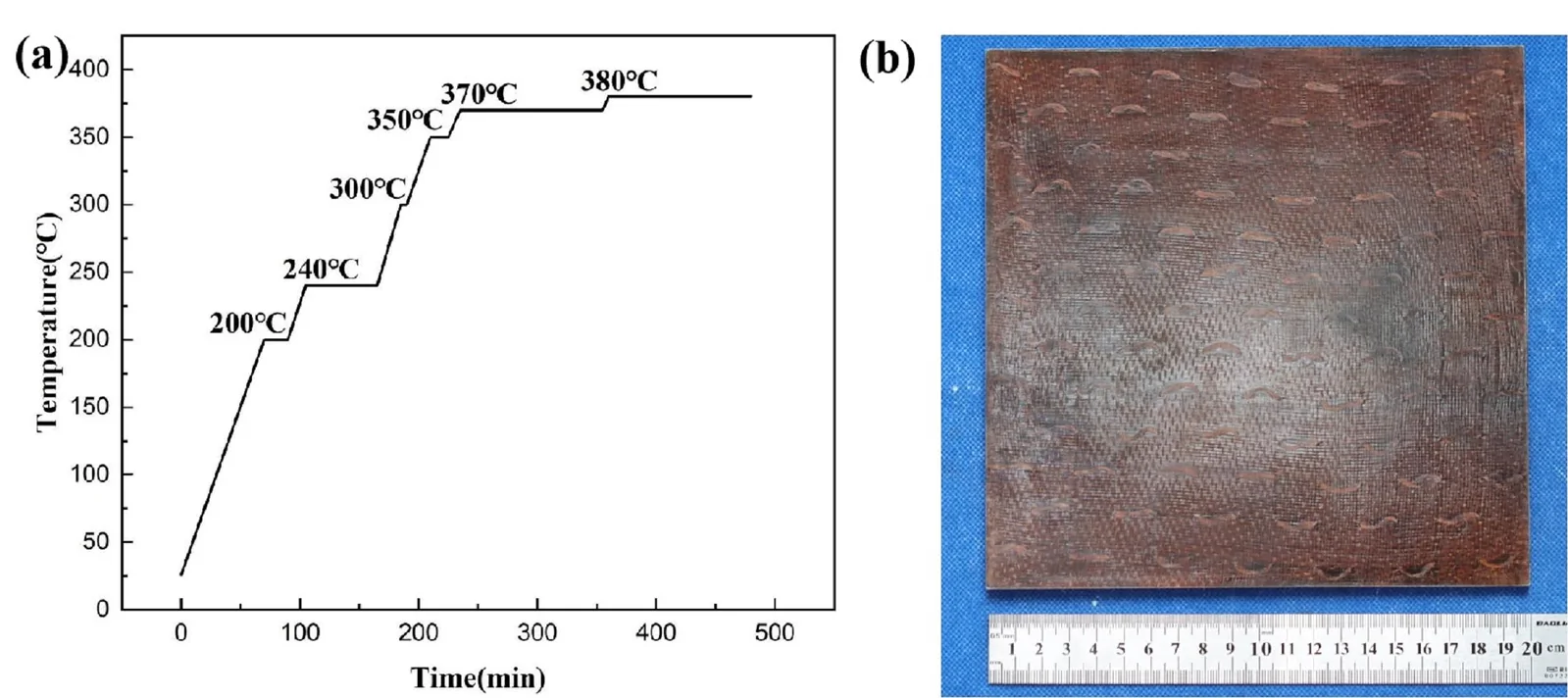
(**a**) Hot-pressing forming process and (**b**) 3D-SiO_2f_/PI composite material after curing.

The 3D quartz-fiber-precursor-reinforced polyimide composite (3D-SiO_2f_/PI) was fabricated though a vacuum-assisted impregnation and hot-press molding method with modified parameters. With the raw materials mentioned above, quartz fiber preforms were manufactured by stitching quartz yarn with a stitch density of 15 mm × 15 mm. Subsequently, the preforms were immersed in alcohol for 48 h to remove the sizing agent on the surface of the fibers. After it was dried thoroughly, the 3D-SiO_2f_/PI prepreg was fabricated through a vacuum-assisted impregnation process. First, the mold surface was cleaned with ethanol and pre-heated to 200 °C for 20 min to activate the release agent. The impregnated preform was then positioned in the mold cavity, and the mold was sealed. The vacuum pump evacuates the air to below 1Pa and then injects the resin solution into the impregnation tank until the resin fully penetrates all the quartz fiber preforms. The impregnation was repeated twice. The first vacuum impregnation lasted for 4 h. After being air-dried for one day, it was placed in a forced-convection drying oven and heated to 100 °C for drying for 3 h. The second vacuum impregnation lasted for 2 h. After being air-dried for one day, it was placed in a drying oven and heated to 100 °C for drying for 5 h. Eventually, the resin gained approximately 75% weight relative to the quartz fiber preforms. The prepreg was cured following the PMR350 resin (solid content 47%, viscosity 190 mPa·s) prepolymer’s fundamental properties [15]. The polyimide prepolymer underwent thermal curing according to the temperature profile shown in Figure 1a. To prevent delamination and void formation, trapped air was evacuated under pressure during the process. The curing cycle proceeded as follows: (1) heating to 240 °C to soften and melt the prepolymer; (2) raising the temperature to 300 °C while applying pressure-assisted degassing, with cycle frequency and duration optimized based on real-time conditions; (3) heating to 350 °C under 8 MPa pressure; (4) further increasing the temperature to 370 °C and maintaining it under pressure for 2 h; (5) heating to 380 °C and holding for another 2 h before cooling. After natural cooling to room temperature, the mold was opened, and the cured composite was removed. Surface grinding was performed to ensure a uniform and smooth edge, as depicted in Figure 1b.

### 2.3. Thermal-Oxidative Aging Treatment

The samples were placed in an air-circulation oven (DHG-9039A, Heheng Instrument & Equipment Co., Ltd. Shanghai, China) for thermal oxygen aging. Two conditions of aging treatment were applied to the 3D-SiO_2f_/PI composites. On one condition, the composites were individually exposed to 350 °C in air for durations of 25, 50, 100, 200, and 300 h, as list in Table 1. On the other condition, the composites were thermal-aged at 250 °C, 300 °C, 350 °C, and 400 °C for 100 h, as list in Table 2.

### 2.4. Characterization

#### 2.4.1. Macroscopic Mechanical Properties Tests

The flexural properties of the 3D-SiO_2f_/PI composite were tested with a universal mechanical testing machine (WDW-50, Kovac Instrument Co., Ltd. Beijing, China) following the standard test method outlined in ASTM D790 [16]. Three-point bending tests were conducted at room temperature with a crosshead speed of 1 mm/min, and the span-to-depth ratio was maintained at 16:1. A minimum of five test specimens were used to ensure statistical reliability of the obtained results.

#### 2.4.2. Microscopic Mechanical Properties Tests

The micromechanical characterization of the 3D-SiO_2f_/PI composite was performed using a nanoindentation system (Hysitron T1950, Bruker Corporation, Germany). Nanoindentation tests were conducted with a Berkovich diamond indenter under displacement control mode (maximum depth: 2000 nm) to determine the elastic modulus and hardness of both the reinforcing fibers and polymer matrix. Furthermore, interfacial properties were evaluated through single-fiber push-in tests using a flat-end cylindrical indenter with a diameter of 5 μm. The interfacial shear strength (IFSS) was calculated based on the maximum de-bonding force using the following equation: *IFSS* = *F*_max_/(*πdl*), where *F*_max_ represents the maximum load at interface failure, *d* denotes the fiber diameter, and *l* corresponds to the embedded fiber length.

#### 2.4.3. Fourier Transform Infrared Spectrum Analysis

Fourier transform infrared (FTIR) spectroscopic analysis was performed using an FTIR spectrometer (Vector-22, Bruker Corporation, Germany) to characterize the chemical functional groups of the materials. Spectra were acquired in transmission mode with a spectral range of 4000–400 cm^−1^ at a resolution of 4 cm^−1^, accumulating 128 scans to ensure an adequate signal-to-noise ratio. For sample preparation, the resin prepolymer powder was thoroughly mixed with spectroscopic-grade potassium bromide (KBr) at a ratio of 1:100 (*w*/*w*) and subsequently pressed into transparent pellets.

#### 2.4.4. Morphology Characterization

The microstructural morphology of specimens was observed by field-emission scanning electron microscopy (MIRA3, TESCAN, Czech Republic).

The pore structure characteristics of the 3D-SiO_2f_/PI composite, including porosity and pore size distribution, were quantitatively analyzed using mercury intrusion porosimetry (Autopore IV9500, Micromeritics Instrument Corporation, USA). The measurements were conducted over a pressure range from 0.1 to 33,000 psi (equivalent to a measurable pore diameter range of 0.006–360 μm).

The three-dimensional microstructure and pore architecture of the 3D-SiO_2f_/PI composites were investigated using X-ray computed tomography (NanoVoxel-3000, Sanying Precision Instruments Co., Ltd., China). The operating voltage and current were set at 200 kV and 200 μA, respectively, with a resolution of 10–12 μm.

The surface chemical composition and elemental distribution of the fiber cloth were characterized using X-ray photoelectron spectroscopy (K-Alpha, Thermo Fisher Scientific, USA). The working voltage during the test process was set to 12 kV, the filament current was maintained at 6 mA, the full spectrum scanning energy was 150 eV, and the step size was 1 eV.

## 3. Results

### 3.1. Porosity Variation and Distribution

**Figure 2:**
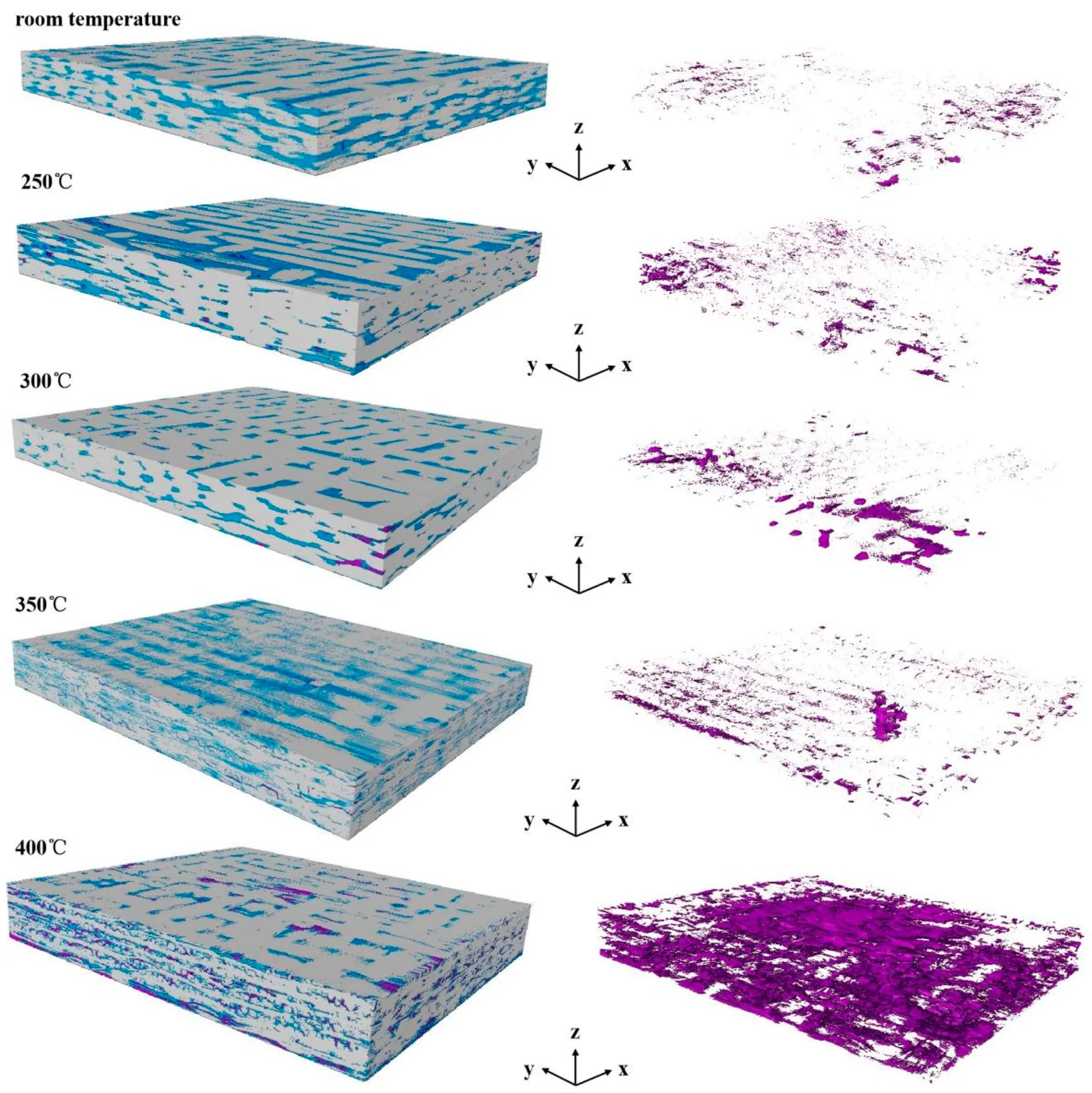
3D morphology of composites with different thermal aging time and temperatures.

Owing to the distinct density differences among quartz fibers, PI resin, and porosity, these components can be effectively differentiated in XCT images through color segmentation. In the three-dimensional reconstructions presented in Figure 2, quartz fibers are represented in white, the resin matrix in blue, and porosity in purple. The reconstructed microstructures reveal that defects are primarily concentrated at the composite surface and surrounding stitch lines during thermal aging at 350 °C for up to 200 h. However, under more severe aging conditions (300 h at 350 °C or 100 h at 400 °C), extensive crack propagation occurs through the inter-laminar regions, resulting in a dense network of inter-connected pores throughout the composite structure.

**Figure 3:**
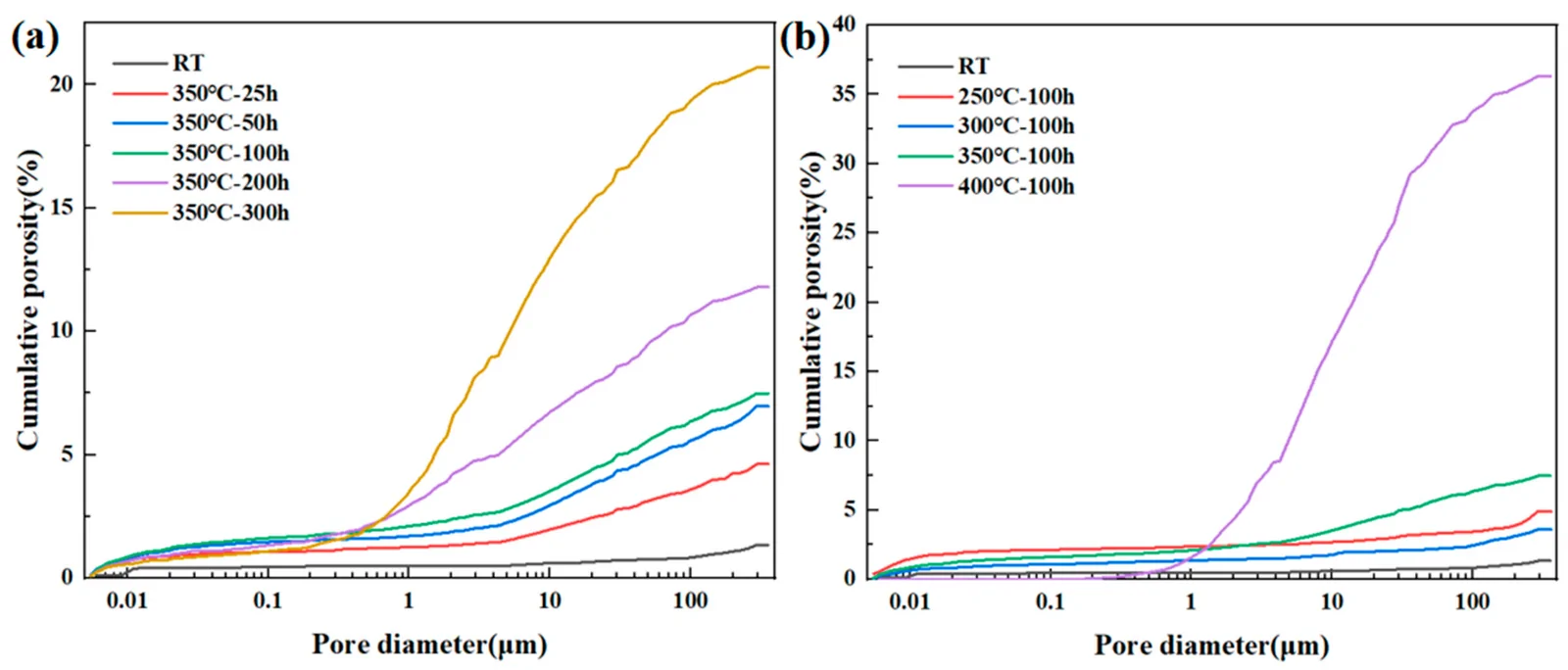
Cumulative pore distribution of 3D-SiO_2f_/PI composite materials at (**a**) different thermal assessment times and (**b**) different thermal assessment temperatures.

To quantitatively assess the porosity-induced defects, a comprehensive characterization approach was employed, combining mercury intrusion porosimetry (MIP) for sub-10 μm pores and X-ray computed tomography (XCT) for pores exceeding 10 μm. This dual-technique analysis provided complete defect distribution profiles for the 3D-SiO_2f_/PI composites, as illustrated in Figure 3. During initial thermal aging stages, the composite exhibits a relatively uniform distribution of pore sizes. However, prolonged thermal exposure leads to progressive resin degradation, resulting in defect growth and crack propagation that inter-connect existing pores. This phenomenon causes a significant increase in the volume fraction of both medium-sized (0.1–10 μm) and large (>10 μm) pores. At lower aging temperatures, the porosity is predominantly characterized by sub 0.1 μm micropores. Elevated temperatures promote crack propagation and micropore coalescence, leading to the formation of larger pores. The porosity distribution statistics of the composite after thermal aging are shown in Table S1. Overall, with the increase in aging temperature, the porosity increases significantly. The porosity after 100 h of aging at 250, 300, 350 and 400 °C was approximately 4.90–36.32%. Notably, at the critical temperature of 400 °C, the composite develops substantial macro-porosity, with 19.09% of the total pore volume consisting of pores larger than 10 μm. This microstructural evolution demonstrates the temperature-dependent nature of defect growth and pore connectivity in the composite material.

**Figure 4:**
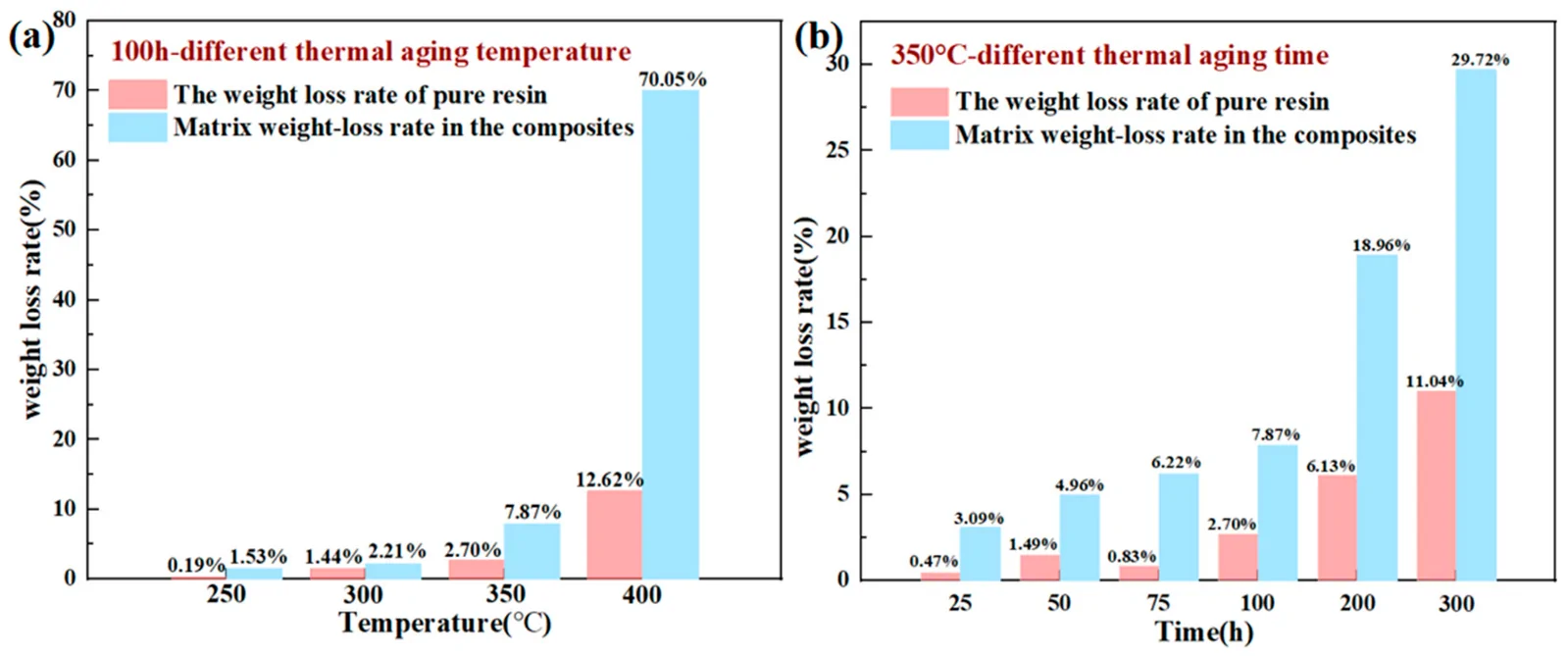
Comparison of matrix loss and pure resin loss rates in composites at (**a**) different thermal assessment times and (**b**) different thermal assessment temperatures.

Figure 4a presents an analysis of weight loss between the matrix in composite materials and pure resin blocks under varying thermal aging durations. The results demonstrate that under the same thermal aging condition, the matrix in composite materials consistently exhibits higher weight loss rates compared to pure resin samples, with the discrepancy progressively increasing over time. This enhanced degradation can be attributed to the increased specific surface area resulting from porosity development, which facilitates more extensive oxidative reactions than those occurring in bulk resin specimens.

Figure 4b illustrates the temperature-dependent weight loss behavior, revealing significantly higher degradation rates in the composite matrix compared to neat resin blocks across all investigated temperatures. In oxygen-containing atmospheres, resin degradation occurs through two concurrent mechanisms: thermal scission of molecular chains and oxidative reactions with atmospheric oxygen. The porous structure of the composite material substantially increases the resin–oxygen contact area, thereby accelerating the oxidative degradation process beyond that in solid resin blocks. This synergistic effect of increased surface area and enhanced oxygen diffusion explains the pronounced difference in degradation rates between the composite matrix and bulk resin samples.

### 3.2. Microscopic Mechanical Properties

**Figure 5:**
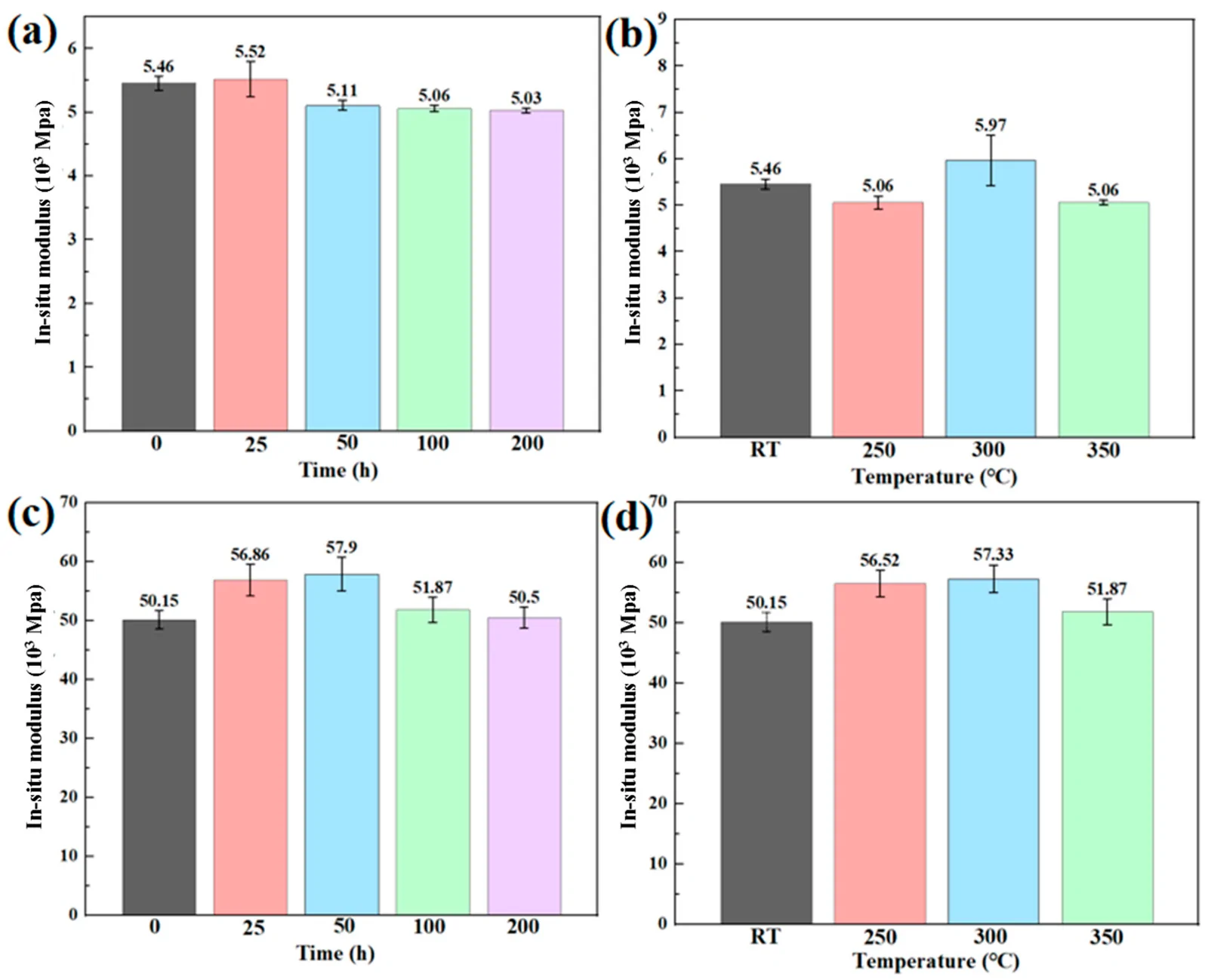
Modulus of 3D-SiO_2f_/PI composite matrix (**a**,**b**) and fiber (**c**,**d**) at different thermal assessment time and temperatures.

As demonstrated in Section 3.1, severe oxidative degradation of the resin matrix occurs in 3D-SiO_2f_/PI composites after 300 h at 350 °C or 100 h at 400 °C, precluding accurate measurement of matrix hardness and modulus under these extreme conditions. Therefore, nanoindentation testing was conducted to determine the in situ modulus of the resin matrix under less severe aging conditions: 25, 50, 100, and 200 h at 350 °C, as well as 100 h at 250 °C and 300 °C. The results indicate that increasing aging temperature has minimal impact on the in situ modulus of PI resin when the aging duration is constant, with measured values fluctuating around 5.36 × 10^3^ MPa. The reinforcing fibers exhibit excellent thermal stability, maintaining a consistent in situ modulus of approximately 53.9 × 10^3^ MPa across all investigated temperatures up to 400 °C. The specific micromechanical parameters of the fibers and the matrix are presented in Figure 5.

The hardness of 3D-SiO_2f_/PI composites was also evaluated by nanoindentation testing. The detailed nanoindentation test curves of the matrix of 3D-SiO_2f_/PI composites at different thermal test temperatures are shown in Figure S1. The hardness test results of the PMR-350 resin matrix after 100 h of thermal aging are listed in Table 3; the hardness of the composite material resin matrix shows an overall stable but slightly increasing trend (except for the samples that have not undergone thermal aging treatment), verifying the good heat resistance characteristics of the resin matrix. The slight growth can be attributed to the less obvious oxidative degradation and the enhanced degree of crosslinking and curing. However, apart from the hardness of the resin matrix, the mechanical strength performance of the composite material still needs to be governed by the fiber/matrix interface. The pyrolysis of the fiber cross-section sizing agent at this temperature might be responsible for the overall strength attenuation of the composite material. It is worth noting that the surface of the sample after thermal aging at 400 °C has been severely oxidized and degraded, and the hardness test cannot be completed, verifying the accelerated oxidation and degradation process after exceeding the critical temperature.

**Figure 6:**
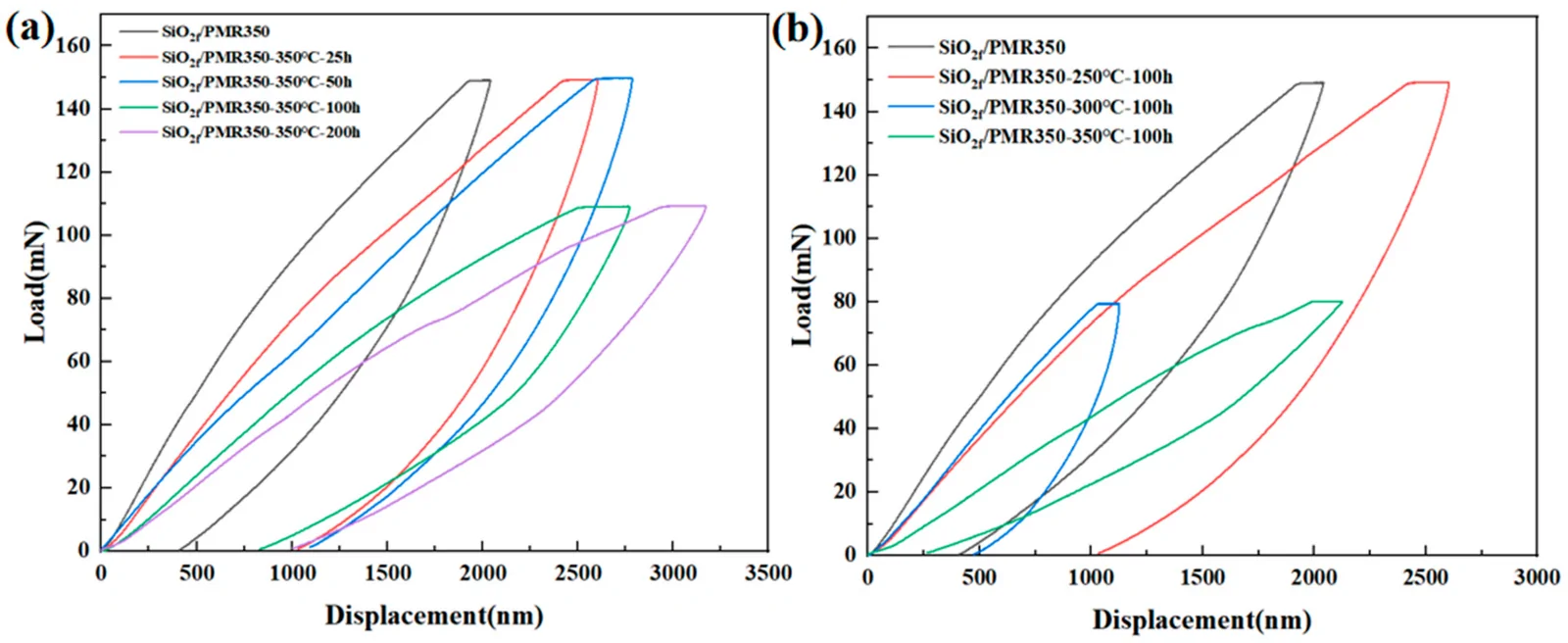
Load–displacement curves of the fiber push-in process of the 3D-SiO_2f_/PI composites at different thermal assessment (**a**) time and (**b**) temperatures.

**Figure 7:**
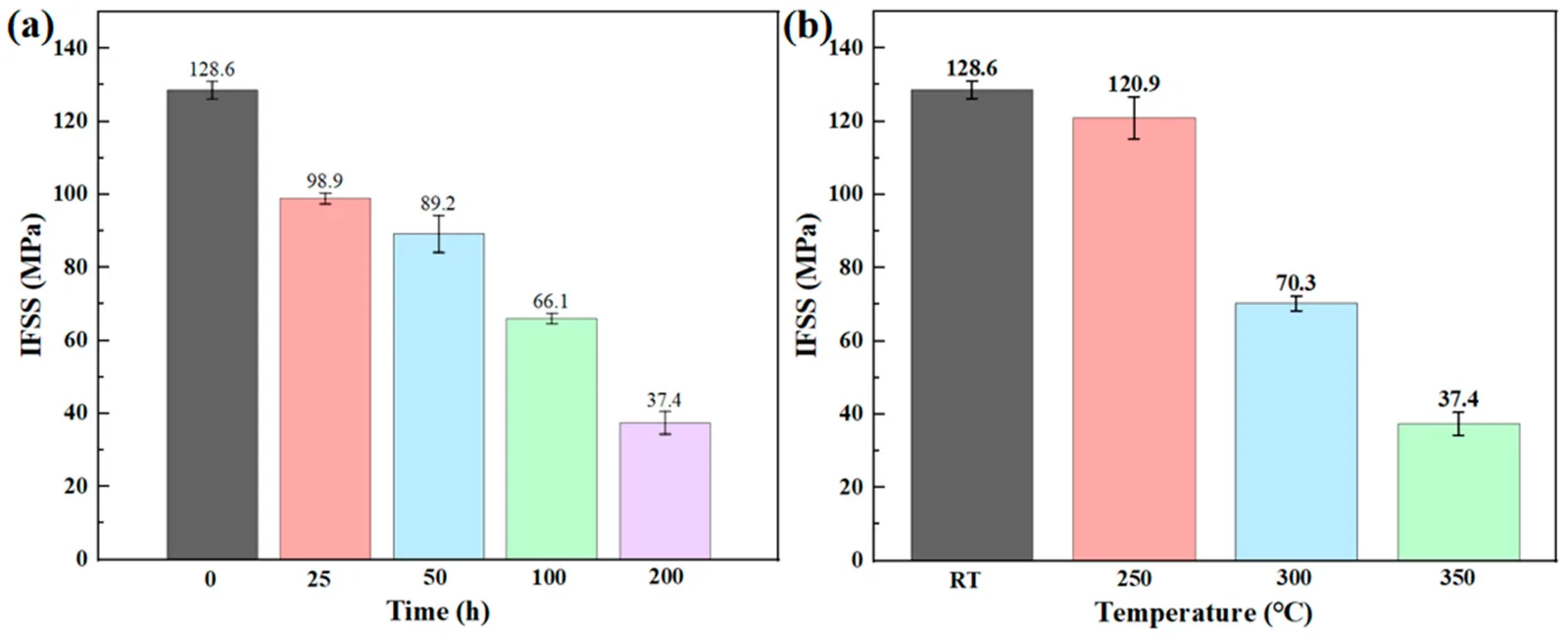
Interfacial shear strength of the 3D-SiO_2f_/PI composites at different thermal assessment (**a**) time and (**b**) temperatures.

The IFSS of composites under different thermal aging conditions was evaluated using single-fiber push-in testing under load control mode. Figure 6 presents the representative load–displacement curves, while Figure 7 summarizes the IFSS values calculated based on the shear-lag model. The experimental results demonstrate a temperature- and time-dependent degradation of interfacial properties. For time-dependent evaluation, the maximum load required for fiber de-bonding progressively decreased with increasing aging duration. At 100 h of aging, the maximum applied load was adjusted to 105 mN to ensure complete fiber de-bonding. All IFSS test curves exhibited characteristic slope transition points, with both the critical load at transition and the curve slope decreasing with prolonged aging, indicating gradual deterioration of interfacial bonding strength. Similarly, temperature-dependent tests revealed reduced maximum loads for fiber de-bonding at elevated temperatures, necessitating an adjustment to 80 mN for effective interfacial failure. Distinct slope transition points were observed in IFSS curves for composites aged at 300 °C and 350 °C. The critical transition load and corresponding slope values showed a progressive decrease with increasing temperature, confirming the temperature-dependent degradation of interfacial shear strength in 3D-SiO_2f_/PI composites.

Based on previous investigations of the PMR350 resin degradation mechanism [15], the thermal-oxidative degradation of polyimide initiates at thermally induced defect sites and subsequently propagates throughout the material matrix. This degradation process exhibits strong temperature dependence, with defects preferentially propagating along the fiber–matrix interface in three dimensions as temperature increases. This interfacial porosity development significantly compromises the IFSS, as the growing voids disrupt stress transfer efficiency and reduce effective bonding area between the reinforcing fibers and the polymer matrix.

### 3.3. Macroscopic Mechanical Properties

**Figure 8:**
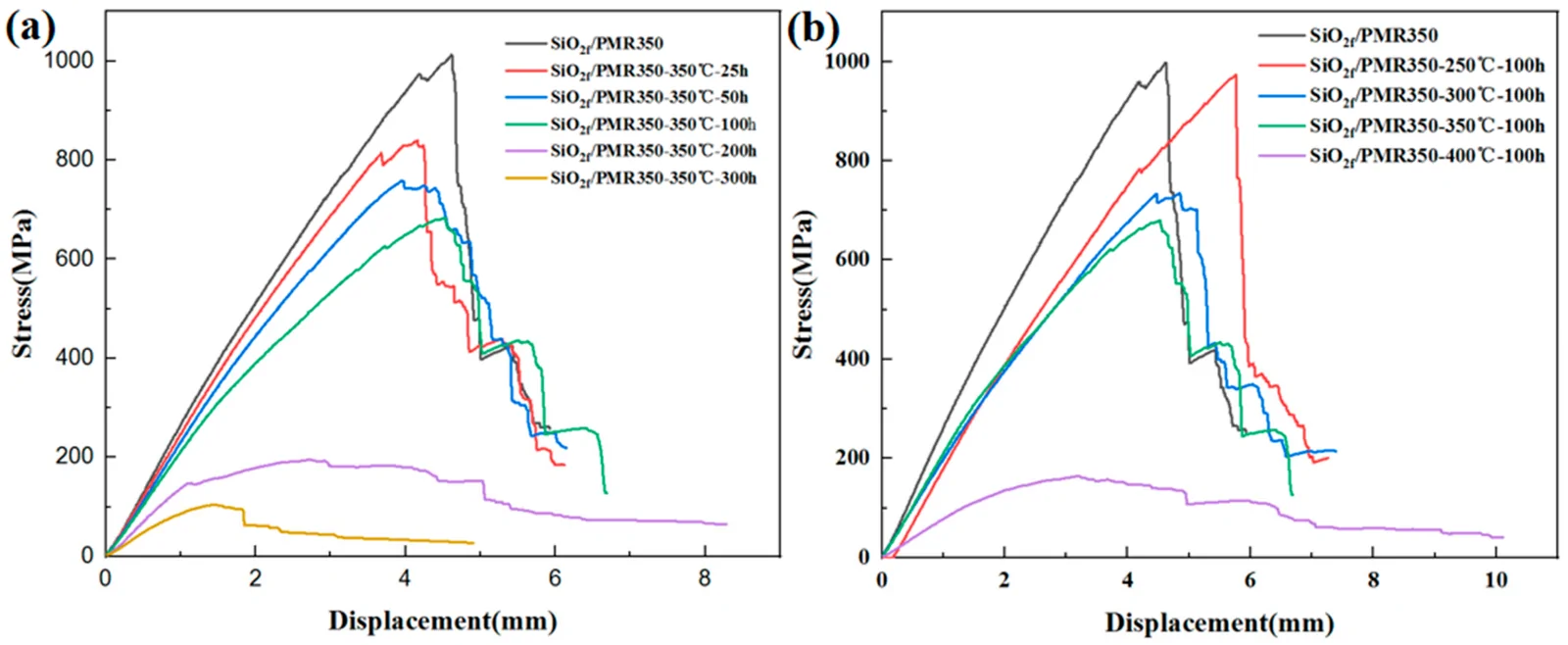
Bending stress–displacement curves of 3D-SiO_2f_/PI composites at different thermal aging (**a**) time and (**b**) temperatures.

**Figure 9:**
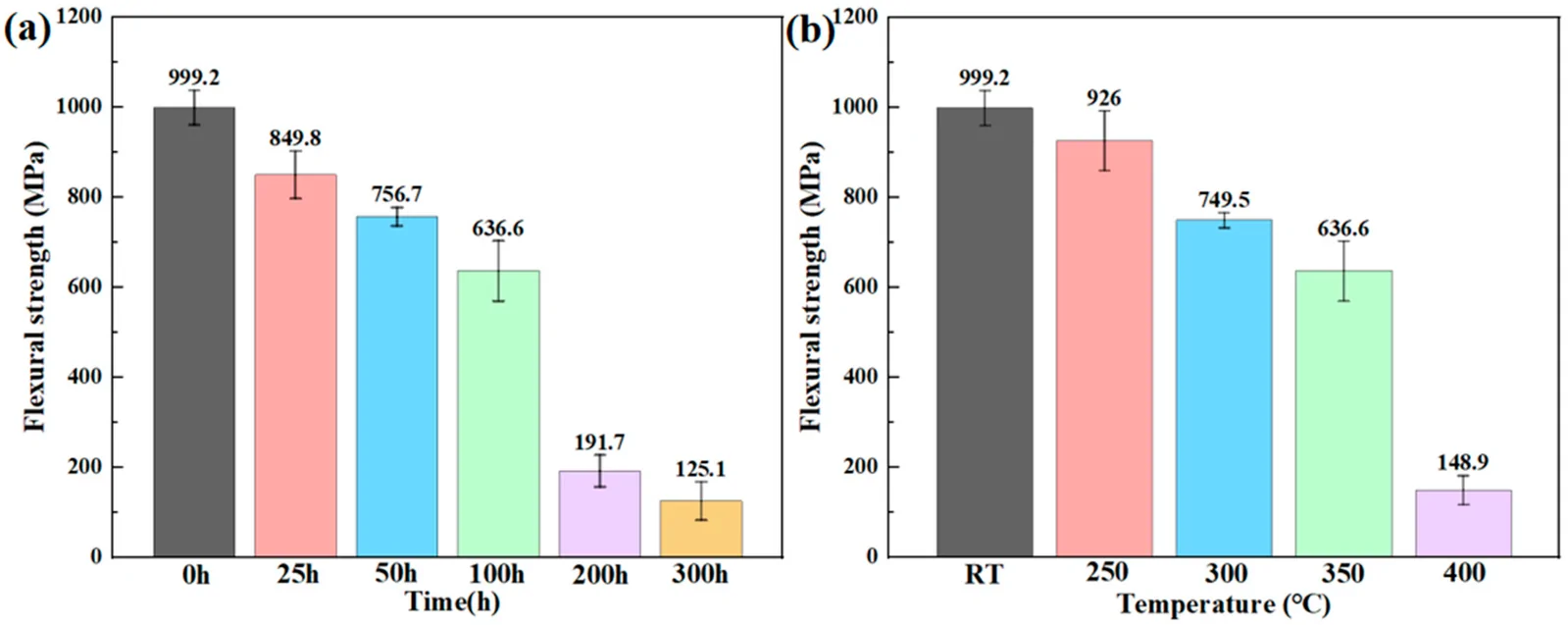
Bending strength of 3D-SiO_2f_/PI composites at different thermal aging (**a**) time and (**b**) temperatures.

A flexural test was conducted for the 3D-SiO_2f_/PI composites. Figure 8a presents the flexural stress–displacement curves of the composites under varying thermal aging durations. The initial slope of the curves for samples aged for 25, 50, and 100 h remains comparable to that of the unaged specimen, indicating maintained stiffness. However, a progressive reduction in maximum load-bearing capacity is observed with increasing aging time. After 200 h of thermal aging, the composite experiences a substantial decrease in flexural strength, retaining only 19.18% of its original strength. This degradation becomes more pronounced after 300 h of aging, with the strength retention rate further dropping to 12.52%, as detailed in Figure 8. Figure 8b reveals that exposure to elevated temperatures (250 °C, 300 °C, and 350 °C) results in a slight reduction in the linear slope of the stress–displacement curves compared to the unaged condition, suggesting a moderate decrease in composite density. This observation is consistent with the measured density reduction in composites aged for 100 h at these temperatures. The maximum load-bearing capacity demonstrates a temperature-dependent decline, becoming particularly significant at 400 °C, where the composite retains only 14.90% of its original flexural strength. Quantitative analysis of flexural strength and modulus under different thermal aging temperatures is summarized in Figure 9.

**Figure 10:**
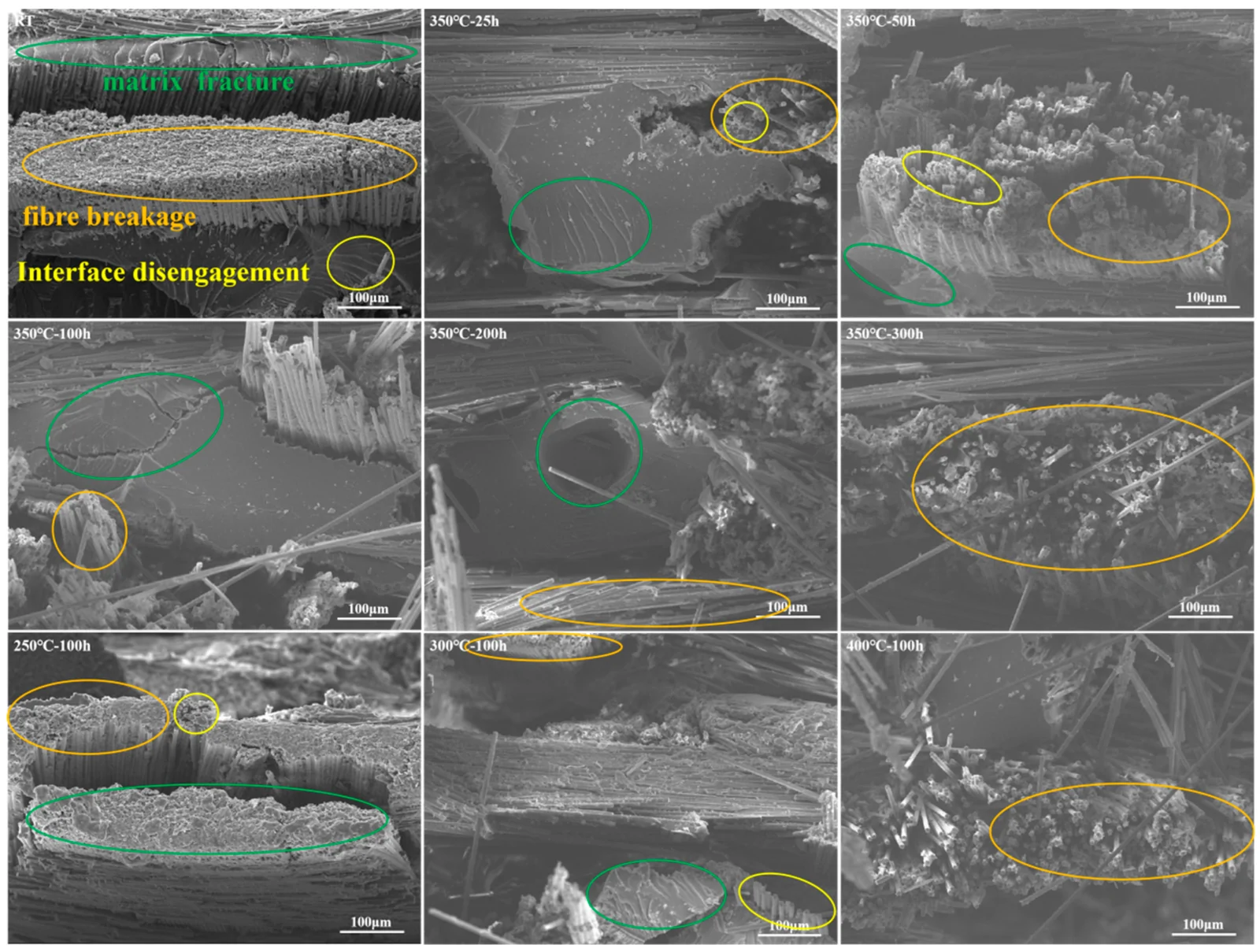
3D-SiO_2f_/PI composite bending fracture SEM for different thermal aging time and temperatures.

Figure 10 presents the microstructural characterization of flexural fracture surfaces, revealing distinct failure mechanisms under various thermal aging conditions. For unaged specimens and those aged for 25 h and 50 h, the fracture morphology demonstrates a combination of three primary failure modes: fiber fracture, matrix cracking, and fiber–matrix interfacial de-bonding. Following 100 h of thermal aging, significant matrix oxidation is observed, accompanied by the formation of microcracks and interfacial gap widening. After 200 h of aging, the fracture surface exhibits characteristic matrix pitting, with fiber fracture becoming the predominant failure mechanism. Specimens aged for 300 h show complete transition to fiber-dominated failure, with no observable matrix contribution. Similarly, specimens aged at 250 °C and 300 °C for 100 h maintain the tri-modal failure mechanism (fiber fracture, matrix cracking, and interfacial de-bonding). However, at 350 °C for 100 h, extensive matrix oxidation leads to pronounced crack formation and significant interfacial gap expansion. The most severe degradation occurs at 400 °C for 100 h, where the matrix is nearly completely degraded, resulting in exclusive fiber fracture as the sole failure mechanism. These observations demonstrate a clear correlation between thermal aging severity and the transition from matrix-dominated to fiber-dominated failure modes.

## 4. Discussion

The experimental results demonstrate that thermal aging parameters significantly influence the material’s porosity, interfacial strength, and flexural properties. From a microstructural perspective, the flexural performance is determined by the synergistic effects of constituent mechanical properties, porosity, and interfacial characteristics. As revealed in Section 3.1, thermal aging parameters exhibit pronounced effects on porosity evolution. Section 3.2 indicates that while the matrix and fiber mechanical properties show limited sensitivity to thermal aging, the interfacial shear strength (IFSS) displays temperature-dependent degradation—exhibiting progressive deterioration with increasing thermal exposure temperature. Consistent with previous findings [11], this interfacial degradation directly contributes to reduced composite fracture strength. Therefore, this section systematically investigates the underlying mechanisms through which thermal aging parameters affect both porosity development and interfacial strength degradation, thereby elucidating their collective influence on the material’s flexural performance.

### 4.1. Thermal Oxidative Degradation of Resin

The extensive literature has established that porosity represents a critical microstructural parameter that substantially influences the mechanical performance of composite materials [17]. The presence of pores not only diminishes the effective load-bearing cross-section but also serves as stress concentration initiators, potentially leading to premature failure. Particularly, the resin matrix and fiber/matrix interfacial regions demonstrate heightened susceptibility to pore-induced damage mechanisms. On a macroscopic scale, the composite material can be conceptually divided into reinforcing fibers, the polymer matrix, and porosity. Based on this simplified model, the total volume of the composite material (*V_c_*) can be expressed as the sum of the fiber volume (*V_f_*), matrix volume (*V_m_*), and pore volume (*V_p_*), as mathematically represented in Equation 1 [18]:

(1) *V*_c_ = *V*_f_ + *V*_m_ + *V*_p_

where *V*_c_ represents the composite volume, *V*_f_ the fiber volume, *V*_m_ the matrix volume, and *V*_p_ the pore volume. During the process of thermal aging, the volume of the fiber remains constant, while the resin matrix gradually degrades with increasing thermal aging temperature, resulting in a decrease in *V*_m_. Consequently, the porosity *V*_p_ keeps increasing. According to the principles of composite mixing, the strength of the composite decreases as *V*_p_ increases.

**Figure 11:**
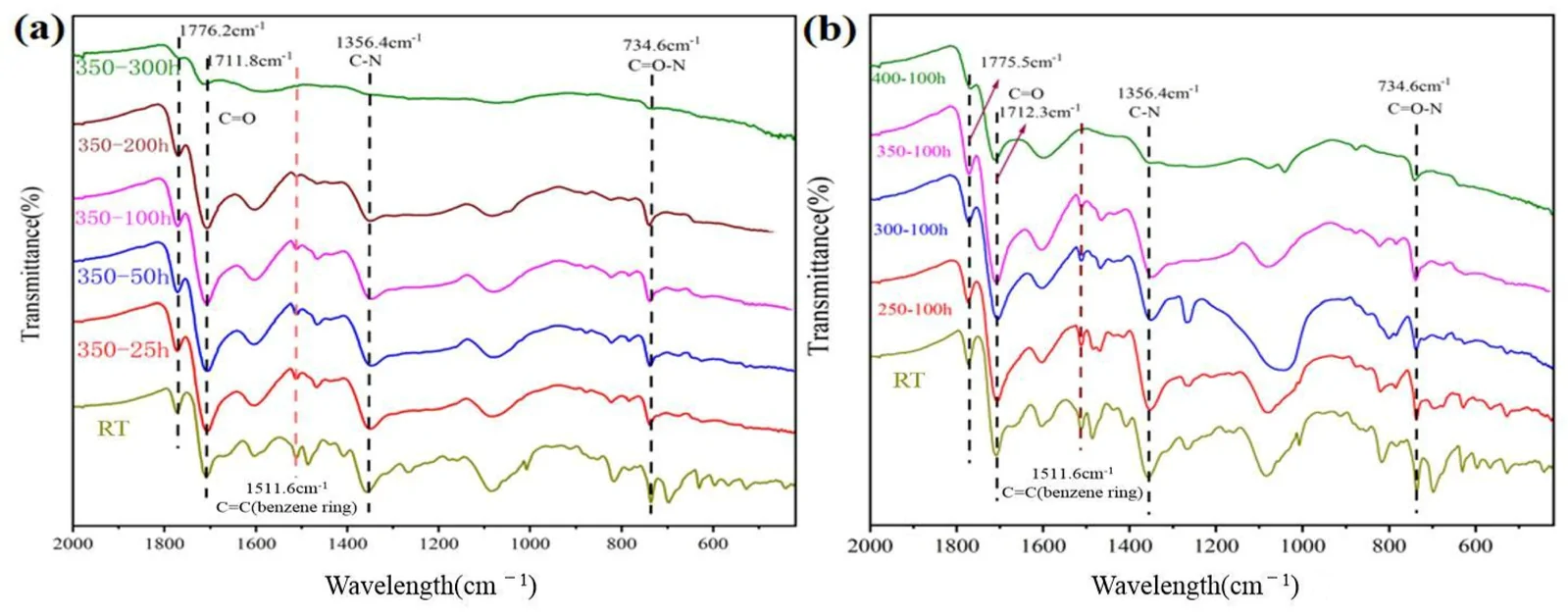
IR spectrum of resins at different thermal aging (**a**) time and (**b**) temperatures.

The primary cause of pore formation in the matrix is the thermal oxidative degradation of the resin. Therefore, the infrared spectrum analysis of PMR350 resin subjected to various thermal assessment time and temperatures is presented in Figure 11. As illustrated, the polyimide resin at room temperature (RT) exhibits five characteristic peaks: the vibration peak of the C=O-N structure at a wavelength of 734.6 cm^−1^, the stretching vibration peak of the C-N structure of the imide ring with a wavelength of 1356.4 cm^−1^, the stretching vibration peaks of the C=O structure at wavelengths of 1712.3 cm^−1^ and 1775.5 cm^−1^, and the vibration peak of the C=C structure in the benzene ring at 1511.6 cm^−1^. With increasing thermal aging time and temperature, the intensity of each characteristic peak gradually diminishes, indicating that the molecular backbone begins to break down. After 200 h of thermal assessment at 350 °C (as shown in Figure 11a), the stretching vibration peak of the C-N structure of the imide ring with a wavelength of 1356.4 cm^−1^ and the vibration peak of the C=C structure in the benzene ring with a wavelength of 1511.6 cm^−1^ have gradually flattened out, and after a thermal assessment at 350 °C for 300 h (in Figure 11a) or at 400 °C for 100 h (in Figure 11b), these characteristic peak essentially disappeared. This suggests that under these thermal assessment conditions, the molecular main chain has fragmented into smaller molecular chains, and the benzene ring has been disrupted.

In fact, the breaking of chemical bonds in polyimide starts from the C-H bond on the benzene ring with lower bond energy, and the breaking of the main chain of the molecule starts from the opening of the C-N bond on the imide ring. With the increase in temperature or the passage of time, the violent decomposition movement on the main chain, such as benzene ring breaking, gradually breaks the molecular chain into smaller segments and free radicals. As the reaction continues, the benzene ring is successively destroyed, and the chemical bonds continue to break, forming many large molecular segments with long molecular chains. Subsequently, these fragments continue to decompose until they are completely broken down into small molecule products. These fragments continue to decompose and react with oxygen, resulting in the generation of small molecular gas products that escape, leading to pore defects in the matrix [19,20,21]. This observation aligns with the previous macroscopic mechanical properties test data. Following the 350 °C thermal assessment for 300 h and 400 °C thermal assessment for 100 h, the bending strength of the composite material was reduced to less than 15% of its original value, indicating that the pore defects resulting from resin degradation significantly impacted the macroscopic mechanical properties of the composite material.

To analyze the role of oxidative degradation in the mechanical property decay of samples, thermogravimetric analysis (TGA) was also employed to evaluate the thermal stability of the samples in both nitrogen and air environments. Figure S2 shows the TGA and DTG curve test results. It can be observed that PMR350 resin, as the main component of the composite material, exhibits a major degradation stage in both nitrogen and air during the thermal degradation process. There is almost no weight loss in the temperature range below 300 °C, and a slow weight loss gradually begins to occur at 300–400 °C. This is because the PMR-350 prepolymer is crosslinked and solidified to form large molecules with conjugated structures. Due to the thermal decomposition temperature of these crosslinked polymer molecules in air being close to 400 °C, they exhibit good thermal stability in the temperature range below 300 °C, regardless of nitrogen or air atmosphere. However, when the temperature approaches or exceeds 400 °C, weight loss rapidly intensifies, and the thermal stability under nitrogen atmosphere is significantly better than that under air atmosphere. Under a nitrogen atmosphere, the 5% thermal decomposition temperature (T_d5%_) is 485 °C, and the residual mass (CR800) at 800 °C is as high as 61.63%. In contrast, under an air atmosphere, the T_d5%_ of the resin decreased to 456 °C, which is 29 °C lower than under a nitrogen atmosphere. At the same time, the maximum weight loss rate temperature also slightly decreased, and the CR800 value almost dropped to zero. Based on the above test results, it can be seen that the thermal degradation of PMR350 resin is faster in an oxygen atmosphere. This is mainly attributed to the presence of oxygen accelerating the thermal degradation process of polyimide resin, resulting in faster molecular chain breakage degradation, a significantly increased weight loss rate, and aggravated mechanical property loss.

### 4.2. Evolution of Interface Damage

**Figure 12:**
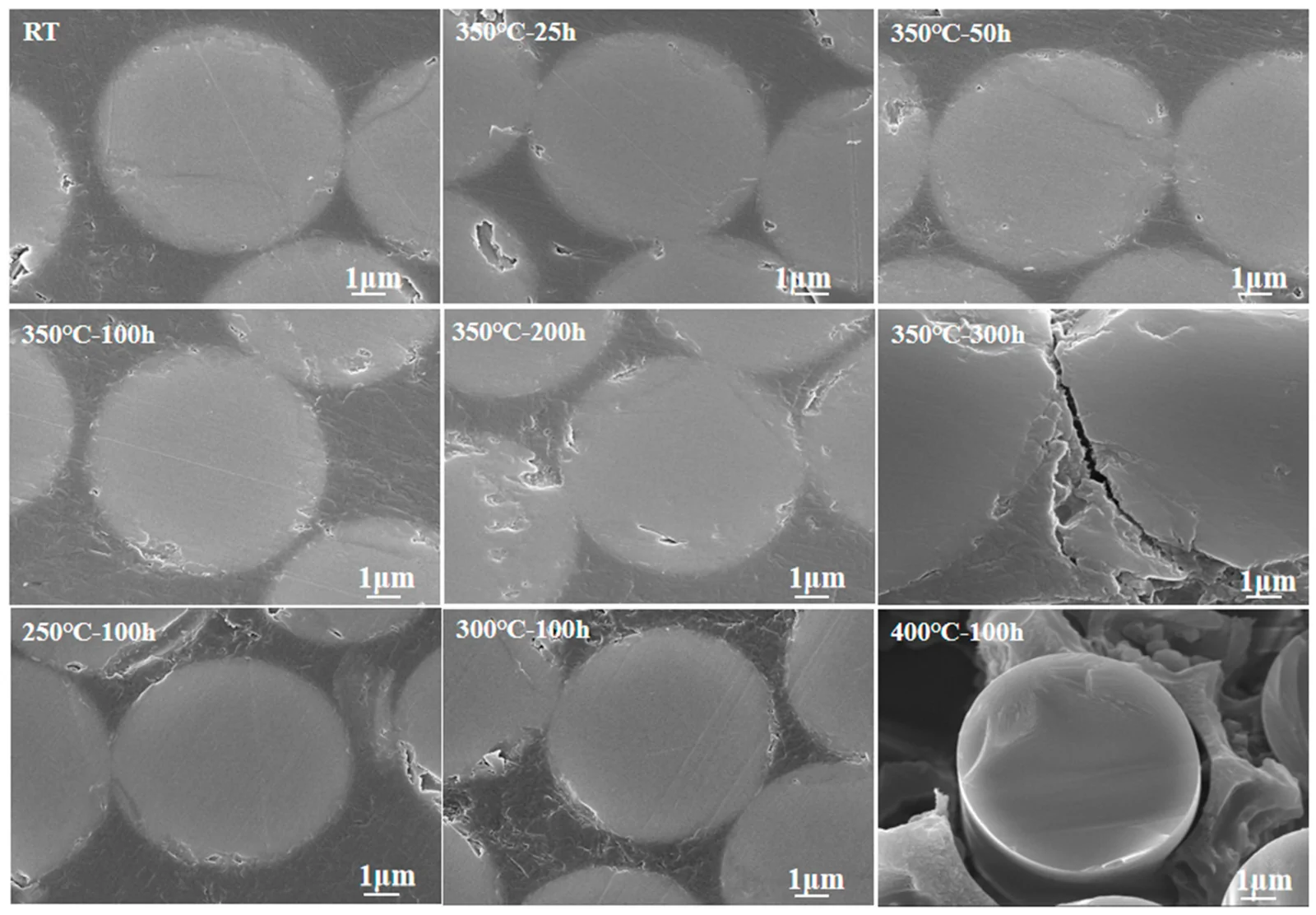
Microscopic morphology of 3D-SiO_2f_/PI composites with different thermal aging time and temperature.

Figure 12 presents the cross-sectional microstructural morphology of 3D-SiO_2f_/PI composites after various thermal aging conditions. When the aging temperature was 350 °C and the aging time was no more than 200 h, the fiber–matrix interface maintained integrity. However, extending the aging duration to 300 h at 350 °C results in significant oxidative degradation of the resin matrix. For composites subjected to 100 h of thermal aging at 250 °C, 300 °C, and 350 °C, the fiber–matrix interface remains well-bonded, with no observable oxidative degradation or thermal decomposition of the resin matrix. The composite structure maintains its integrity, showing no evidence of porosity or crack formation. In contrast, the 3D-SiO_2f_/PI composite aged at 400 °C for 100 h exhibits severe matrix degradation. While the fiber cross-sections maintain their circular morphology, the near-complete absence of resin matrix between adjacent fibers indicates extensive oxidative degradation at this elevated temperature. This microstructural evolution demonstrates the critical temperature and aging time threshold beyond which the composite experiences catastrophic matrix degradation.

**Figure 13:**
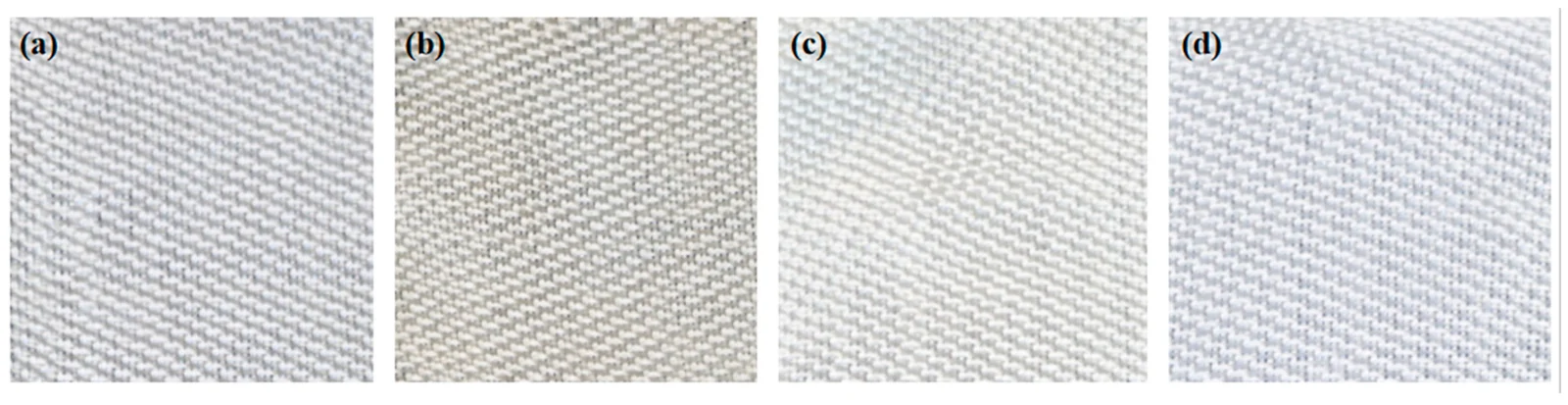
Fiber cloth surface change (**a**) before (RT), (**b**) during (240 °C) and (**c**) after (250 °C, 0 h) the curing cycle, and (**d**) after thermal aging (250 °C, 100 h).

The characterization of the micromorphology of the composite material in Figure 12 indicates that, in the absence of overheating aging, the defects in the composite material are primarily located at the junction of the fiber bundles and the matrix, particularly at the periphery of the suture line, where some cracks have become quite evident. In order to investigate the defects of the contact surface of the fiber during the curing process of the matrix, the composite curing process of the quartz fiber cloth was examined after the removal of the sizing agent. As illustrated in Figure 13, the fiber exhibited distinct color transitions corresponding to different thermal stages: pristine white color at room temperature, characteristic yellowing upon reaching 240 °C, and gradual fading of the yellow tint upon completion of the curing cycle. Subsequent thermal aging at 250 °C for 100 h resulted in complete restoration of the original white coloration. These colorimetric changes provide visual evidence of thermal-oxidative reactions occurring at the fiber surface, reflecting the complex interplay between thermal degradation and oxidative processes during different stages of thermal exposure.

**Figure 14:**
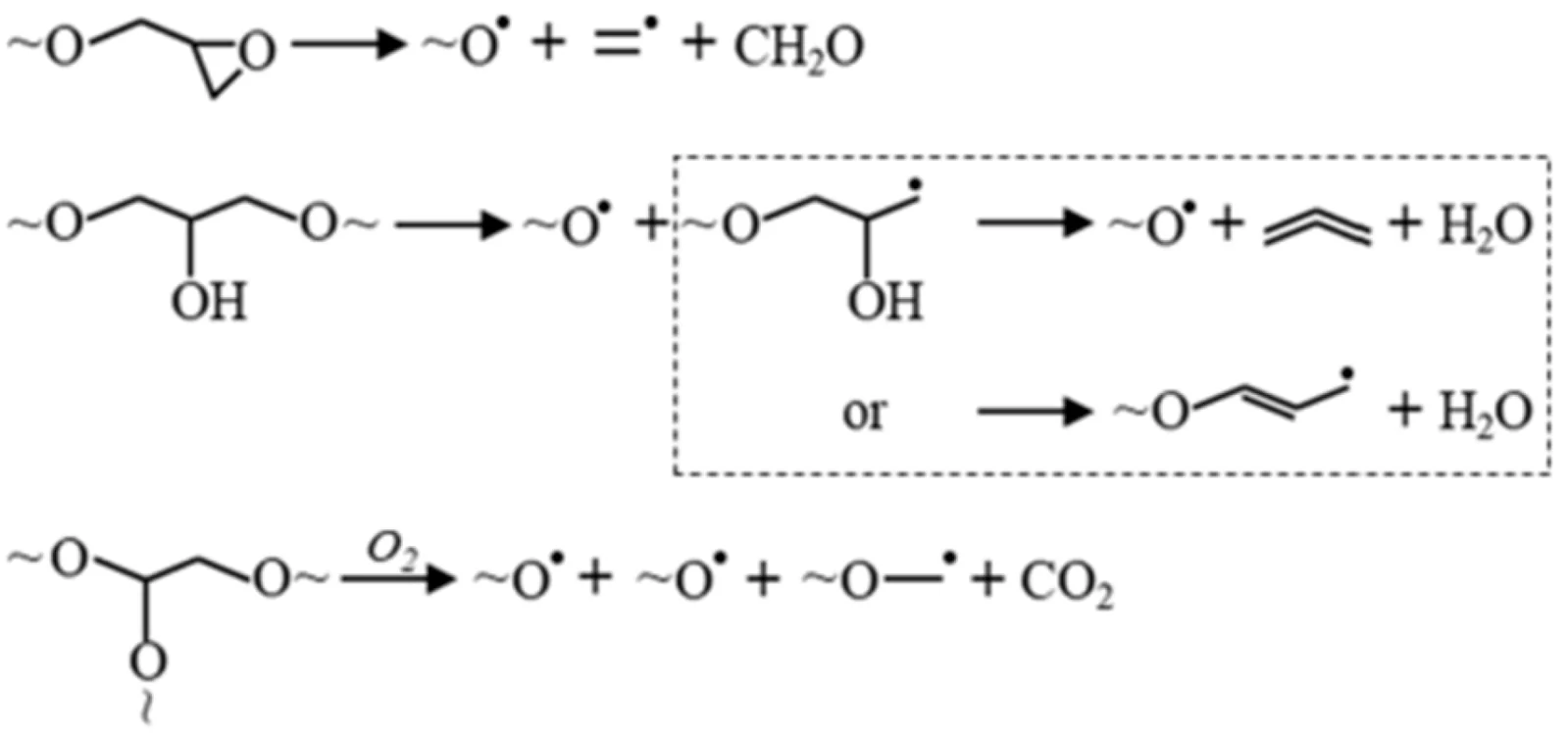
Typical thermal decomposition process [23] of the main group in the epoxy resin.

The sizing agent on the fiber surface, primarily composed of epoxy resin, undergoes significant oxidative degradation in oxygen-rich environments. The glycidyl ester ether and amine groups within the epoxy molecules are particularly susceptible to oxidation, leading to the formation of chromophoric groups that cause the characteristic yellowing of the resin surface [22]. Prolonged exposure to oxygen leads to a reaction between the epoxy resin and oxygen, generating H_2_O, CO_2_ (as illustrated in Figure 14), and other gaseous carbon oxides, while hydroxides escape from the fiber surface.

**Figure 15:**
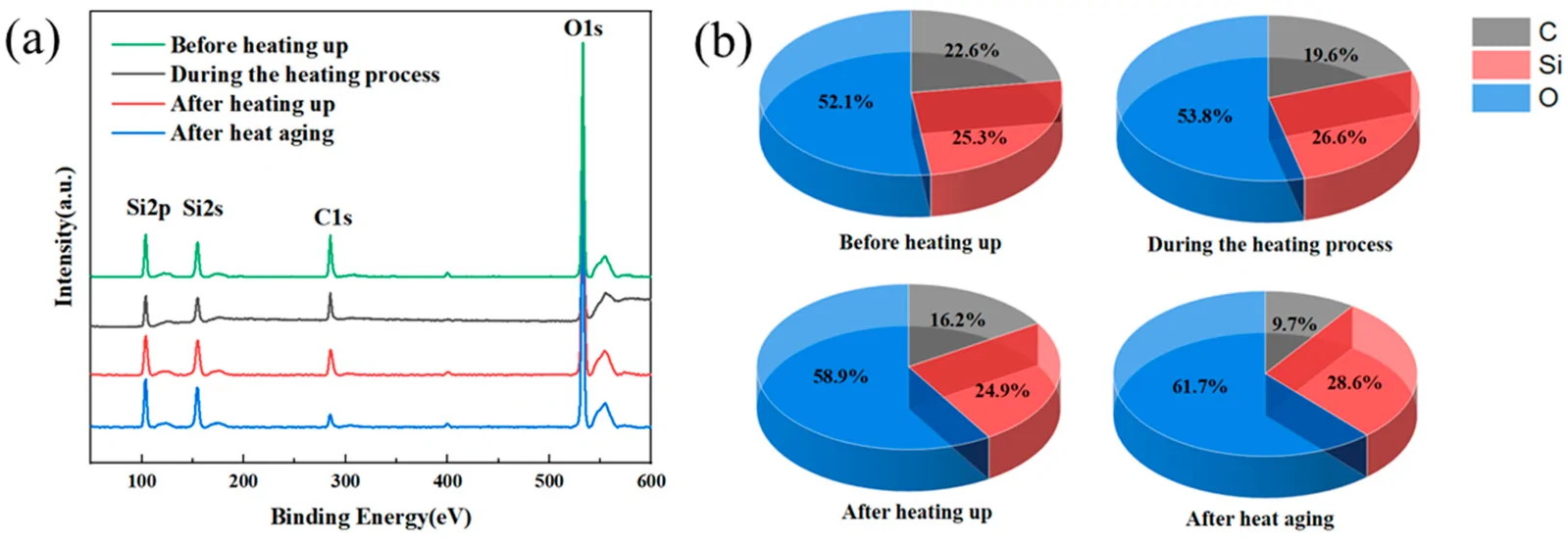
The XPS spectra survey (**a**) and relative element content in the quartz fibers (**b**).

As shown in Figure 15a, XPS characterization was performed on quartz fibers at three distinct stages: pre-curing, during curing, and post-curing after 100 h of thermal aging at 250 °C. The analysis revealed a progressive decrease in carbon peak intensity across these stages. Complementary observations from Figure 13 and Figure 15b indicate that the fiber surface maintains a light yellow coloration after the curing process but reverts to its original white color following 250 °C/100 h thermal aging. The correlation between the color transition of quartz fiber fabric and the corresponding elemental composition changes in XPS spectra aligns with the thermal decomposition characteristics of epoxy resin. These findings demonstrate that the residual sizing agents on the fiber surface undergo partial decomposition during the curing process and are nearly completely decomposed after 100 h of thermal aging at 250 °C.

**Figure 16:**
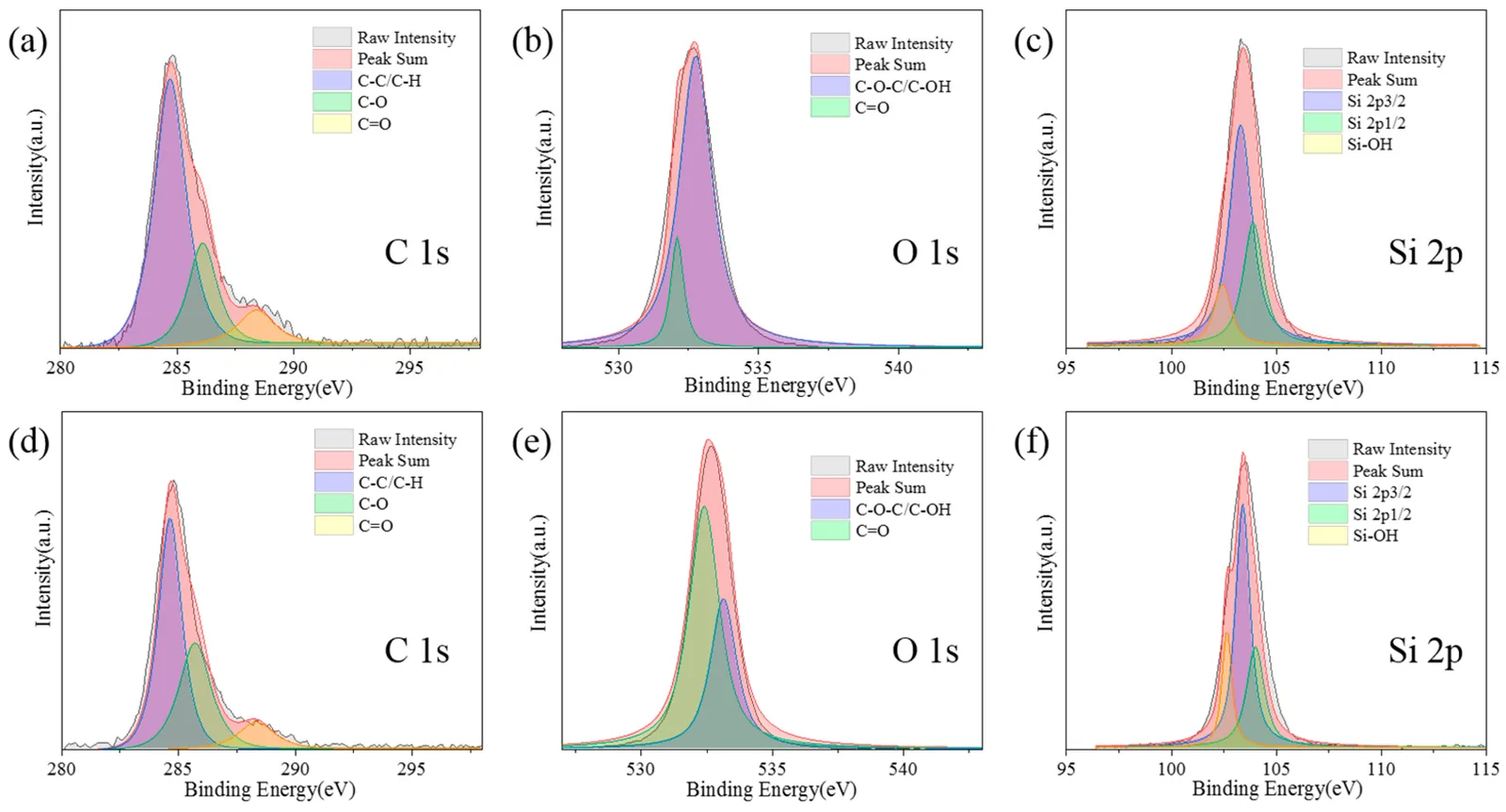
High-resolution deconvoluted XPS spectra for C, O, and Si before (**a**–**c**) and after (**d**–**f**) thermal aging.

High-resolution XPS spectra deconvolution was carried out to investigate the chemical states of C, O, and Si before and after thermal aging. As shown in Figure 16, the high-resolution C 1s spectrum was fitted to three types of C functional groups: benzene ring and methyl group (C-C/C-H), epoxy group (C-O), and carbonyl groups (C=O), with carbon atoms centered at 284.7, 286.1, and 288.4 eV, respectively. Here, C-C/C-H and C-O were attributed to the epoxy resin, confirming the presence of sizing agent on the fiber surface, while the C=O group was assigned to the oxidation of the sizing agent. Correspondingly, the O 1s spectrum was fitted to characteristic peaks of the epoxy skeleton/hydroxyl groups (C-O-C/C-OH) and the carbonyl group (C=O) at 532.8 and 532.1 eV, respectively. The Si 2p spectrum was fitted to a pair of 2p1/2-2p3/2 doublets centered at 103.9 and 103.3 eV, and a Si-OH peak at 102.5 eV. After thermal aging, the C=O characteristic peak area significantly enhanced in the C 1s and O1s spectra, especially in the O1s spectrum, as well as the Si-OH peak in the Si 2p spectrum, confirming the decomposition of residual sizing agent after 250 °C/100 h thermal aging.

**Figure 17:**
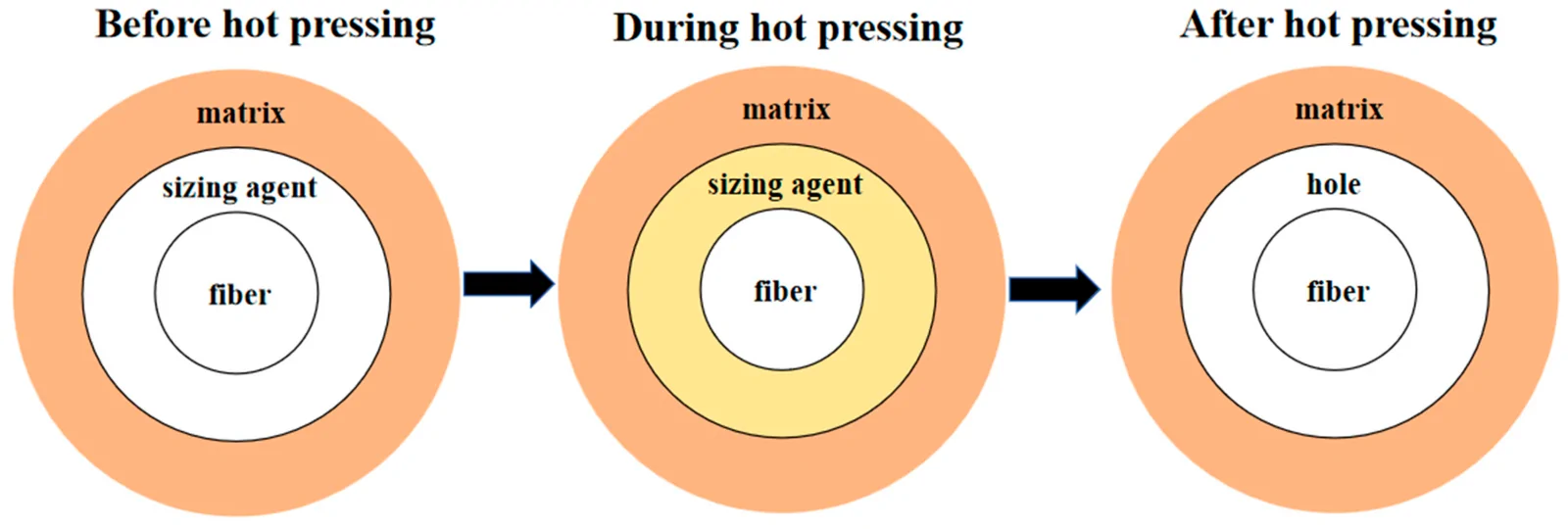
Interfacial evolution due to the oxidation of the sizing agent.

The incomplete removal of fiber sizing agents following 48 h of ethanol immersion represents the fundamental cause of porosity formation between the interface of fiber bundles and the resin matrix during the hot-pressing process. Residual sizing agents undergo thermal decomposition and subsequent oxidation reactions at elevated temperatures, leading to gas evolution and void formation at the fiber–matrix interface. The evolution of the interface between the fiber and the resin matrix is illustrated in Figure 17.

**Figure 18:**
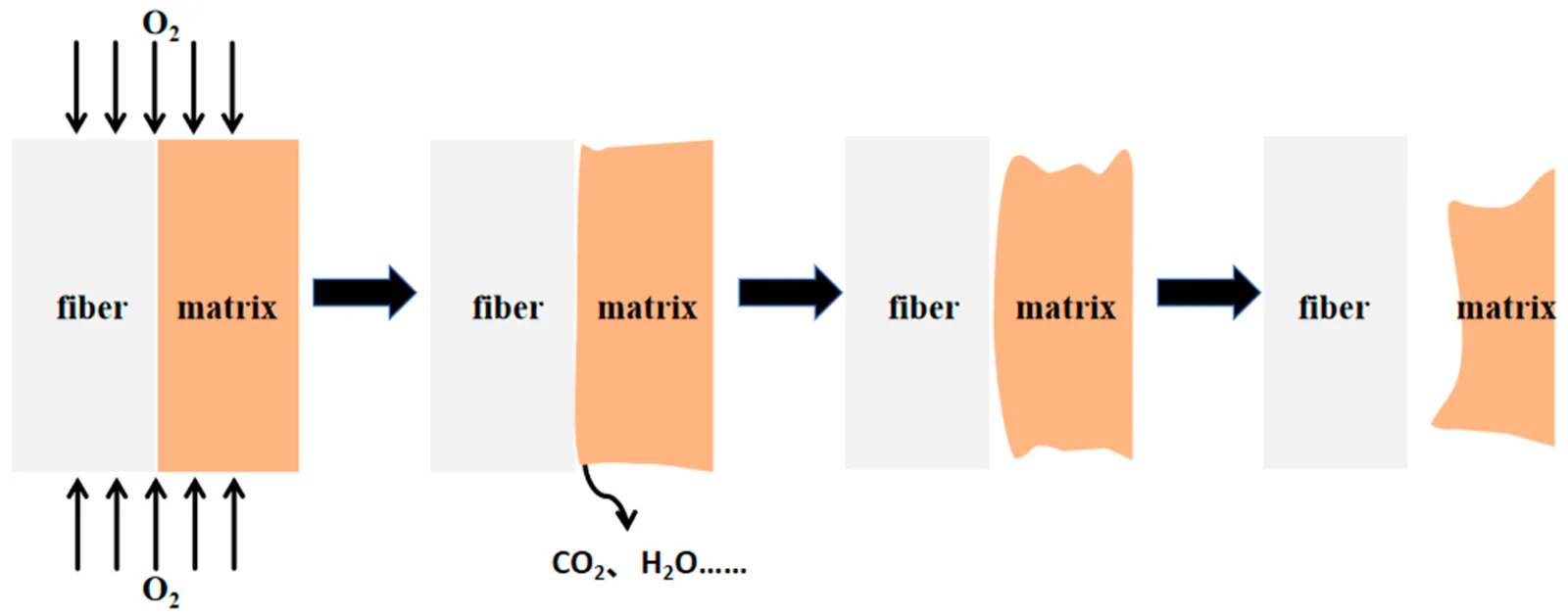
Interface evolution during thermal aging.

With increasing thermal aging temperature, the polyimide resin undergoes molecular chain scission, accompanied by oxidative degradation in the presence of oxygen. This dual degradation mechanism leads to the generation of gaseous carbon oxides and hydroxides, significantly accelerating the resin decomposition process. Pre-existing microcracks at the fiber–matrix interface provide enhanced pathways for oxygen diffusion, thereby increasing the reactive surface area and facilitating progressive interfacial degradation. The synergistic effect of thermal decomposition and oxidative degradation results in accelerated deterioration of the fiber–matrix interface, as schematically illustrated in Figure 18.

Based on the analysis of PMR350 degradation mechanisms, the thermal aging process within the temperature range of 250–400 °C represents the initial stage of thermal decomposition. The degradation initiates at thermally induced defect sites and progressively propagates throughout the material matrix. As the thermal exposure intensifies, the resin degradation propagates radially from these defect sites, leading to extensive matrix deterioration. This progressive matrix degradation results in a substantial reduction in IFSS between the reinforcing fibers and resin matrix, primarily due to the loss of interfacial integrity and stress transfer capability.

### 4.3. Comparison of Mechanical Properties After Thermal Aging

Limited research has been conducted on mechanical properties of SiO_2f_/PI composites after thermal aging. Table 4 and Table 5 present a comparison of flexural strength and IFSS, respectively. This work systematically investigated the influence mechanisms of thermal aging time and temperature on the bending strength and IFSS of SiO_2f_/PI composites. As displayed in Table 4, the room-temperature flexural strength of SiO_2f_/PI composites in this work surpasses literature-reported values, and favorable strength retention is achieved after 100 h of thermal aging. Similarly, the IFSS of SiO_2f_/PI composites obtained in this work is higher than values reported in the literature, and a satisfactory strength retention is maintained after 100 h of thermal aging, as shown in Table 5.

## 5. Conclusions

In this article, long-term high-temperature thermal aging testing was carried out for 3D-SiO_2f_/PI composites, and comprehensive characterization of microstructural and mechanical property degradation in 3D-SiO_2f_/PI composites was performed. The effects of thermal aging temperature on the structural integrity and mechanical performance of 3D-SiO_2f_/PI composites were systematically investigated. The IFSS of 3D-SiO_2f_/PI composites exhibited significant temperature-dependent degradation after 250 °C to 400 °C isothermal aging for 100 h, with complete interfacial failure occurring at 400 °C. Flexural strength also showed similar deterioration, retaining less than 15% of its original value after aging at 400 °C for 100 h. On the other hand, 350 °C isothermal aging for 300 h led to a progressive decrease in IFSS from 128.6 MPa to near-zero. The flexural strength also exhibited comparable degradation. The mechanical degradation mechanism was primarily attributed to severe thermal oxidative degradation of the polyimide matrix and the intrinsically linked interfacial degradation. Thermal oxidation within the resin phase induced pore formation and propagation. At the same time, oxygen diffusion through developed porosity accelerated progressive oxidative degradation from surface to bulk, subsequently compromising fiber–matrix interfacial bonding. The synergistic effects of matrix degradation and interfacial de-bonding collectively contributed to the reduction in composite mechanical performance. These findings establish that the thermo-oxidative stability of the resin is the critical determinant governing porosity development and IFSS of 3D-SiO_2f_/PI composites.

## Figures and Tables

**Table 1:** The experimental design for thermal aging test with different aging duration.

Temperature (°C)	Time (h)
350	25
350	50
350	100
350	200
350	300

**Table 2:** The experimental design for thermal aging test with different aging temperature.

Temperature (°C)	Time (h)
250	100
300	100
350	100
400	100

**Table 3:** The hardness of 3D-SiO_2f_/PI composite at different thermal aging temperatures.

Temperature (°C)	Hardness (GPa)
RT	0.49 ± 0.02
250	0.42 ± 0.01
300	0.44 ± 0.01
350	0.50 ± 0.01
400	—

**Table 4:** Comparison of flexural strength after thermal aging.

Ref.	Thermal Temperature	Thermal Aging Time, 0 h	Thermal Aging Time, 50 h	Thermal Aging Time, 100 h	Thermal Aging Time, 200 h
[13]	230 °C	709	664	660	628
[12]	300 °C	615	655	524	/
This work	250 °C	999	/	926	/
	300 °C	999	/	749.5	/
	350 °C	999	756.7	636.6	191.7

**Table 5:** Comparison of IFSS after thermal aging.

Ref.	Thermal Aging Temperature	Thermal Aging Time, 0 h	Thermal Aging Time, 50 h	Thermal Aging Time, 80 h	Thermal Aging Time, 100 h	Thermal Aging Time, 200 h
[14]	300 °C	102	/	86.54	/	/
	400 °C	102	/	41.64	/	/
This work	250 °C	128.6	/	/	120.9	/
	300 °C	128.6	/	/	70.3	/
	350 °C	128.6	89.2	/	66.1	37.4

## Data Availability

The data supporting the findings of this study are available from the corresponding author upon reasonable request. The data are not publicly available due to [Data are contained within the article].

## Supplementary Materials

The following supporting information can be downloaded at: https://www.mdpi.com/article/10.3390/polym18202556/s1, Figure S1: Matrix nanoindentation load-displacement curves of 3D-SiO_2f_/PI composites at different heat test temperatures (a) modulus variation curves (b); Figure S2: TGA and DTG test result of PMR-350 matrix of 3D-SiO_2f_/PI composite. Table S1: The porosity distribution statistics of the composite after thermal aging.
