# Supplementary material for: Investigation on the Degradation Mechanisms and Mechanical Property Evolution of 3D-SiO2-Fiber-Reinforced Polyimide Composites After Thermal Aging
Source: Polymers (Basel). 2026 Oct 17;18(20):2556. doi: 10.3390/polym18202556 (PMC13709274; doi:10.3390/polym18202556)
Supplement: Supplementary file 1 [file polymers-18-02556-s001.zip › polymers-4600526-supplementary.pdf]

Supplementary information

Table S1. The porosity distribution statistics of the composite after thermal aging.

Temperature(°C)	Porosity by volumetric method	Porosity by mercury intrusion method		
	Cumulative porosity(%)	Pore size (<0.1μm,%)	Pore size (0.1~10μm,%)	Pore size (>10μm,%)
RT	1.34	0.45	0.15	0.74
250	4.90	2.12	0.58	2.20
300	3.60	1.08	0.68	2.84
350	7.49	1.62	1.93	3.94
400	36.32	1.72	15.51	19.09

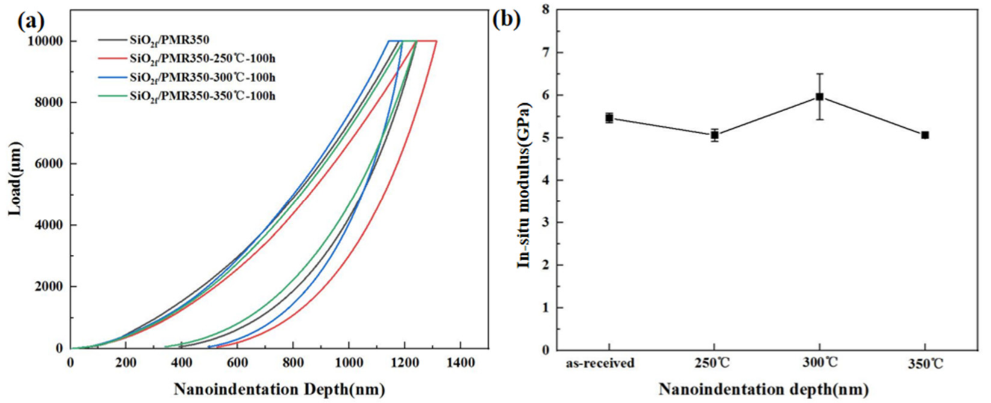

Figure S1. Matrix nanoindentation load-displacement curves of 3D-SiO_{2f}/PI composites at different heat test temperatures (a) modulus variation curves (b)

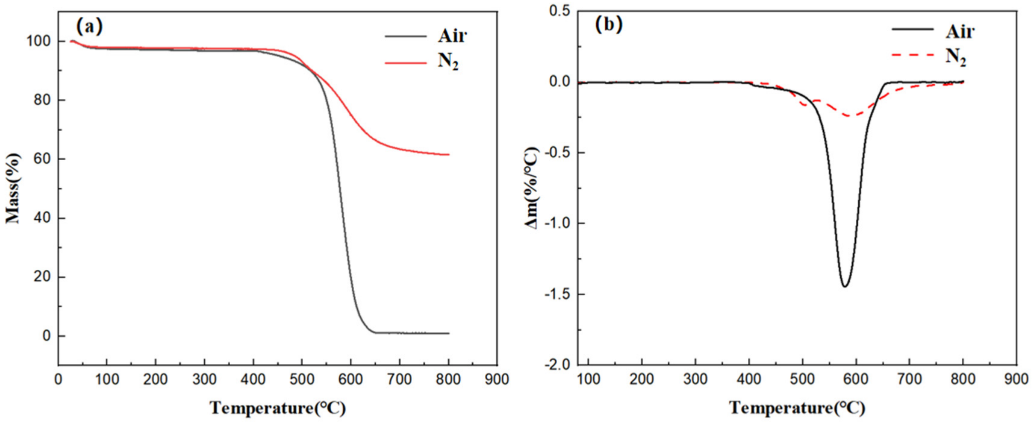

Figure S2. TGA and DTG test result of PMR-350 matrix of 3D-SiO_{2f}/PI composite
